# RETRACTION: Quinovic Acid Impedes Cholesterol Dyshomeostasis, Oxidative Stress, and Neurodegeneration in an Amyloid‐β‐Induced Mouse Model

**DOI:** 10.1155/omcl/9789810

**Published:** 2026-06-19

**Authors:** Oxidative Medicine and Cellular Longevity

RETRACTION: K. Saeed, S. A. Shah, R. Ullah, et al., “Quinovic Acid Impedes Cholesterol Dyshomeostasis, Oxidative Stress, and Neurodegeneration in an Amyloid‐β‐Induced Mouse Model,” *Oxidative Medicine and Cellular Longevity* (2020): e9523758, https://doi.org/10.1155/2020/9523758.

The above article, published online on 21 November 2020 in Wiley Online Library (wileyonlinelibrary.com), has been retracted by agreement between the Journal Editor‐in‐Chief, Jeannette Vasquez Vivar; and Wiley Periodicals LLC. Concerns were raised by Elizabeth Bik on PubPeer [1] that the Control and A beta panels in Figure 2E appear to show the same sample. The publisher confirmed this concern. The authors responded by providing their raw image data, but their responses to the concerns regarding image duplication were unsatisfactory. Because the images appear to be manipulated, the Editors have lost confidence in the results published. As a result, the data and conclusions are considered unreliable. Therefore, the article must be retracted.
